# Sensitivity Mapping of a Terahertz Split-Ring Resonator Metasurface for Local Microplastic Detection in Water: Effects of Particle Position and Shape

**DOI:** 10.3390/ma19173790

**Published:** 2026-09-06

**Authors:** Adam Ruszczynski, Michal Herbko, Przemyslaw Lopato

**Affiliations:** Center for Electromagnetic Fields Engineering and High-Frequency Techniques, Faculty of Electrical Engineering, West Pomeranian University of Technology in Szczecin, ul. Sikorskiego 37, 70-313 Szczecin, Poland; adam.ruru@gmail.com (A.R.); przemyslaw.lopato@zut.edu.pl (P.L.)

**Keywords:** terahertz sensing, split-ring resonator (SRR), metasurface, microplastics, sensitivity map, dielectric sensing, water monitoring, particle shape

## Abstract

Conventional vibrational spectroscopy provides chemically specific identification of microplastics but often requires extensive sample preparation and particle-by-particle analysis. This study numerically examines a terahertz split-ring resonator (SRR) metasurface as a transducer for local dielectric perturbations in water. A finite-element model was used to quantify the spatial response of the active gap. A 5 × 5 sensitivity map yielded frequency shifts of 0.37–0.97 GHz for equivalent local polystyrene perturbations, with maxima at the gap corners, consistent with simulated field localization. An equal-volume shape study compared a spherical inclusion (Δ*f* = 4.34 GHz) with ellipsoids of identical volume and constant height; shifts of 4.25–4.56 GHz demonstrated dependence on in-plane orientation. At the highest-sensitivity cell, shifts of 1.12, 0.97, 0.69, 0.25, and 0.24 GHz were obtained for PE, PS, PET, and two synthetic high-permittivity references, respectively. For three four-corner configurations, the observed shifts agreed with reference-cell estimates within 1.4%. The results identify positional, dielectric, and geometric factors that should be controlled in future microfluidic preconcentration and hybrid-sensing experiments.

## 1. Introduction

Micro- and nanoplastic contamination of aquatic environments is a recognized global concern, with plastic fragments reported in surface waters, groundwater, wastewater, and drinking water, and with documented pathways into food chains and consumer products [1,2,3,4,5]. Reliable monitoring of these particles remains analytically challenging because of their small size, irregular morphology, chemical diversity, and the complexity of environmental matrices [1].

Established laboratory workflows based on micro-FTIR and Raman microspectroscopy provide chemically specific identification of polymer particles but commonly require filtration, sample cleaning, controlled imaging, and particle-by-particle spectral interpretation [6,7]. These constraints limit sample throughput and motivate the development of complementary, rapid screening approaches that could act as a first-pass indicator of dielectric loading in a water sample before full spectroscopic analysis; emerging sensor technologies are increasingly discussed in this role [8].

Terahertz (THz) technology offers one such route [9]. Metamaterials and metasurfaces composed of subwavelength metallic resonators [10,11,12] enable strong local field confinement and have been developed into a broad family of THz sensing devices [13,14], including microfluidic platforms for dielectric characterization of liquids [15] and ultrasensitive resonant sensors [16]; recent reviews summarize this progress [17]. Split-ring resonators (SRRs) are particularly relevant because their capacitive gap concentrates the electric field into a small active volume [18], which has been exploited for detecting micro-scale dielectric targets such as microorganisms [19] and polystyrene microbeads [20]. Notably, experimental work has shown that the resonant response of THz metamaterials depends on the shape of deposited polystyrene microbeads, with non-spherical particles producing larger resonance shifts than spherical ones of comparable size [20], and that the sensitivity of a metamaterial gap is spatially non-uniform, as demonstrated by selective surface functionalization [21]. Surface-functionalized microfluidic THz metamaterials have recently been applied to the detection of polystyrene microplastic particles directly in water [22].

The present work builds on the terahertz metasurface research line of our group, which includes THz inspection of polymer composites [23,24], numerical and experimental analysis of fabrication-uncertainty effects on THz metasurface resonances [25], the influence of thin dielectric layers on square-SRR metasurface resonances [26], and position-tunable Jerusalem-cross metasurfaces for thin-film evaluation [27].

Compared with the experimental studies of references [20,21,22], which addressed dry or surface-deposited targets and probed sensitivity distribution indirectly through functionalization, this article provides a systematic numerical characterization of a single SRR metasurface configuration operating directly in a water environment. The study: (i) presents the sensor geometry and its transmission response in air and in water; (ii) maps the frequency-shift response on a 5 × 5 grid spanning the active gap; (iii) quantifies, at constant inclusion volume, the influence of particle shape by replacing a spherical polystyrene inclusion with prolate and oblate ellipsoids; (iv) compares single-site responses of dielectric loadings representing PE, PS, PET, and two synthetic high-permittivity references; and (v) tests whether multi-site loadings are approximately additive. The aim is not to claim chemically selective microplastic identification but to establish a numerical basis for particle positioning, preconcentration strategies, and future experimental validation.

## 2. Materials and Methods

### 2.1. Sensor Design and Operating Principle

**Figure 1:**
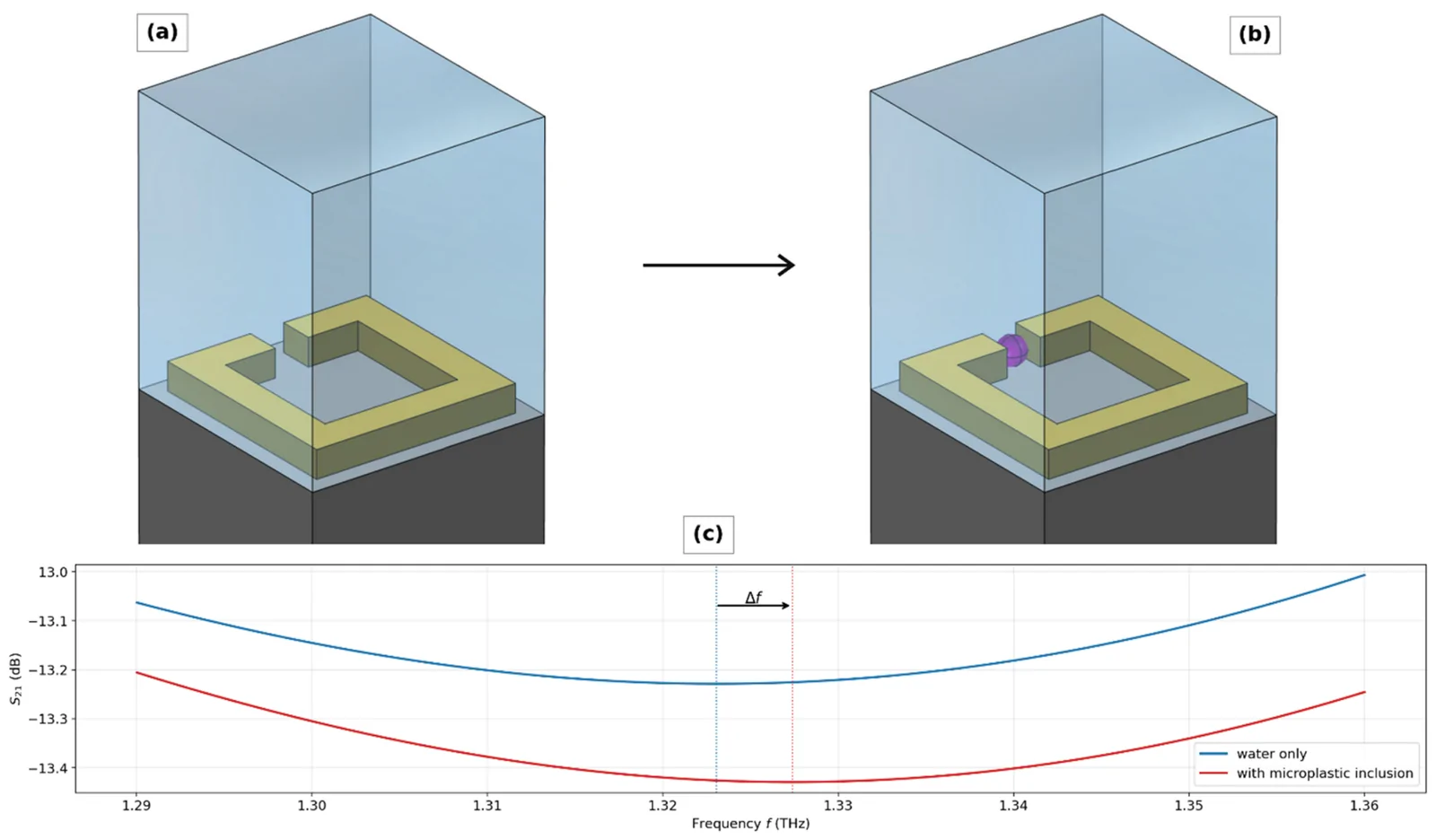
Operating principle of the SRR microplastic sensor: (**a**) unit cell with the domain above the resonator filled by water only; (**b**) unit cell with a local microplastic inclusion in the gap region; (**c**) simulated transmission characteristics *S*_21_(*f*) for both states (spherical inclusion, *r* = 1 µm), showing the resonance shift Δ*f*. In panel (**c**), the blue curve denotes the water-only reference and the red curve the PS-loaded state; the dashed vertical lines mark the corresponding resonance minima, and Δ*f* denotes their frequency separation.

**Figure 2:**
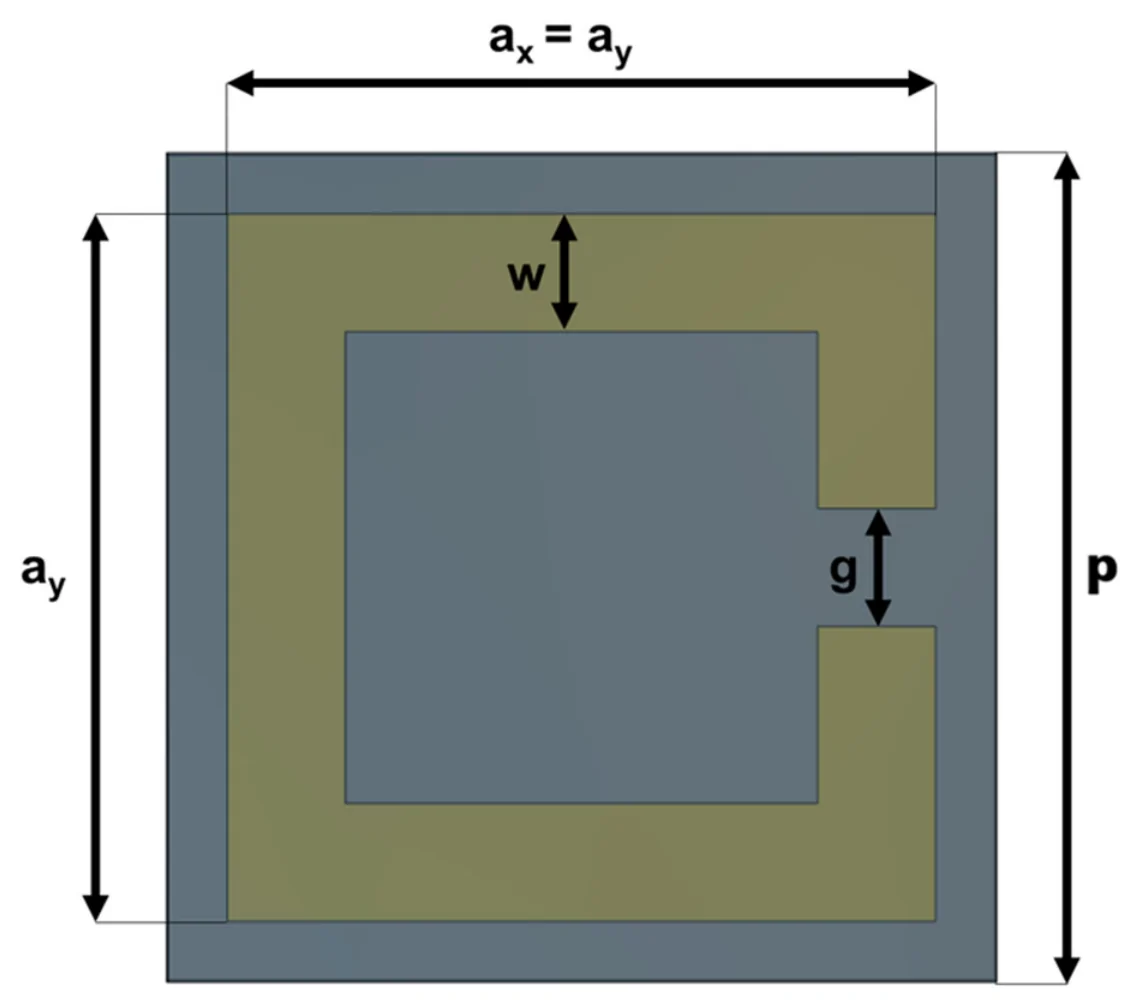
Geometry of the unit cell (top view) with dimensions *a*_x_ = *a*_y_ = 15 µm, with a ring width *w* = 2.5 µm, a gap width *g* = 2.5 µm, and a unit-cell period *p* = 17.5 µm.

The analyzed sensor is a periodic metasurface whose unit cell contains one square gold split-ring resonator (SRR) fabricated on a silicon substrate with a thin titanium adhesion layer operated in transmission with a water domain above the resonator plane. The final configuration considered throughout this article has a resonator side length of *a*_x_ = *a*_y_ = 15 µm and a gold layer thickness of *h*_Au_ = 2.00 µm, with a trace width *w* = 2.5 µm, a gap width *g* = 2.5 µm, and a unit-cell period *p* = 17.5 µm. The silicon substrate thickness is 520 µm, the titanium adhesion-layer thickness is 5 nm, and the water domain above the resonator extends 20 µm. The operating principle is illustrated in Figure 1, and the dimensions are annotated in Figure 2.

For the fundamental mode of operation, the equivalent-circuit of SRR constitutes an *LC* resonator with inductance *L* of the ring and capacitance *C* concentrated in the gap. The resulting resonant frequency is given as:

(1) $$f_{0}=\frac{1}{2\pi \sqrt{L\cdot C}}$$

(*V*_PS_) denotes the volume of the polystyrene inclusion within the active gap region, whereas (*V*_gap_) is the effective field-active gap volume; therefore, the ratio *V*_PS_/*V*_gap_ acts as a filling factor, with a larger value producing a greater capacitance reduction and, consequently, a larger blue-shift:

(2) $$\frac{\Delta C_{eff}}{C_{eff}}\approx \frac{V_{PS}}{V_{gap}}\frac{\varepsilon_{PS}-\varepsilon_{med}}{\varepsilon_{med}}$$

A local dielectric perturbation inside the gap modifies the effective capacitance (*C*_eff_). In a first-order perturbation description, because the relative permittivity of polystyrene in the analyzed band (*ε*_PS_ ≈ 2.5) is lower than the real part of the permittivity of water (*ε*_med_), the local replacement of water by polystyrene reduces the effective gap capacitance (*C*_eff_) and shifts the resonance (*f*_0_) toward higher freq. (blue-shift, Δ*f* > 0)

(3) $$\frac{\Delta f}{f_{0}}\approx -\frac{1}{2}\frac{\Delta C_{eff}}{C_{eff}}$$

### 2.2. Numerical Model

**Figure 3:**
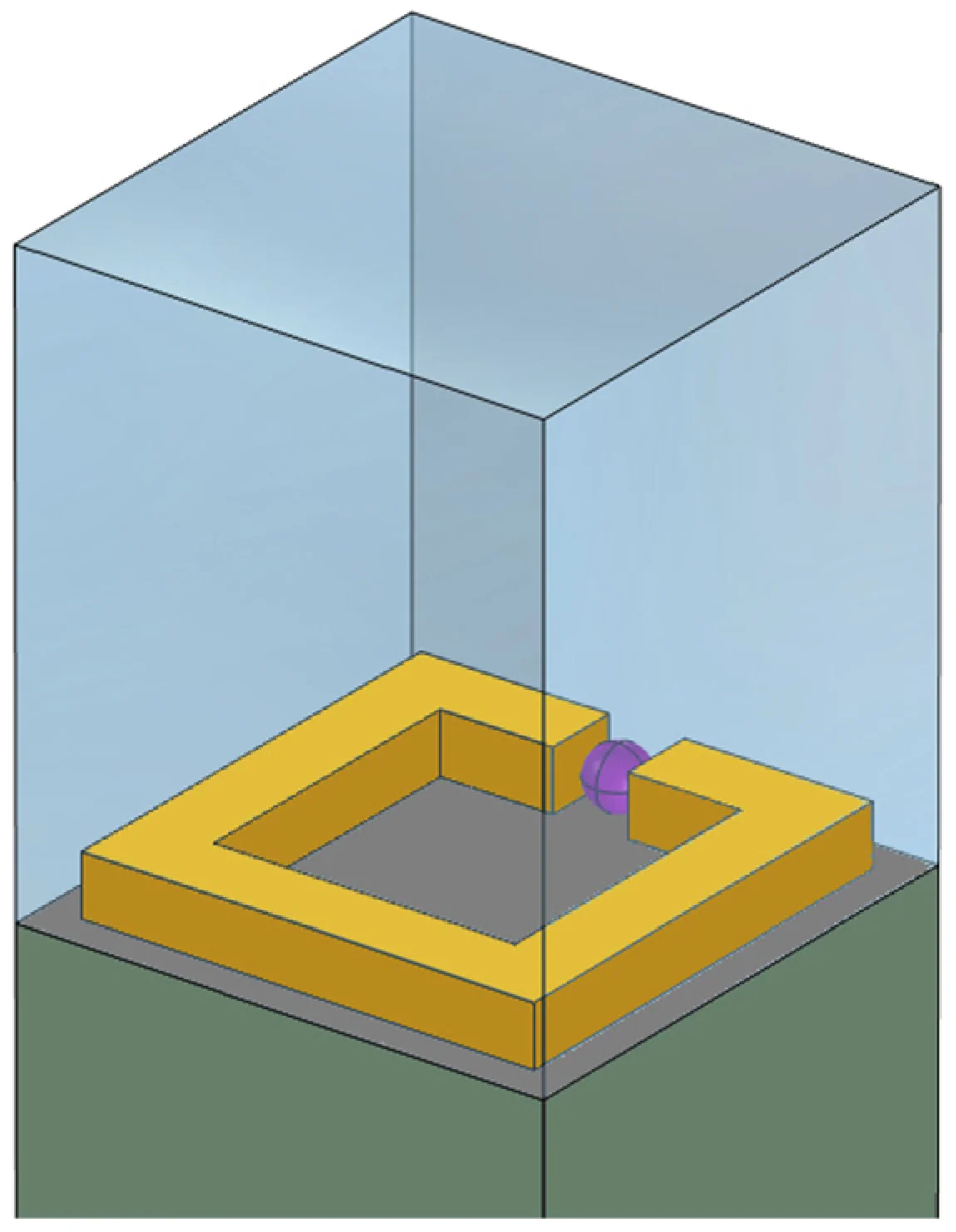
COMSOL unit-cell model: silicon substrate, Ti/Au split-ring resonator, water domain, and local gap inclusion. In the rendering, the silicon substrate is shown in green, the Ti/Au resonator in gold/yellow, the water domain in transparent light blue, and the local inclusion in purple.

Frequency-domain finite-element simulations were performed in COMSOL Multiphysics® version 6.4 (COMSOL AB, Stockholm, Sweden) using the RF Module [28]. The computational domain is a periodic unit cell excited at normal incidence with Floquet (periodic) ports, and the electric field is polarized perpendicular to the gap-bearing side of the resonator. Floquet periodic boundary conditions are applied on the lateral walls of the unit cell to reproduce the infinite periodic metasurface. The sensor response is evaluated from the transmission parameter *S*_21_(f). The material description is summarized in Table 1. The complete three-dimensional numerical model, including the water domain and the local gap inclusion, is shown in Figure 3. Water was modeled as a dispersive medium with a frequency-dependent complex relative permittivity based on the reference data of Ellison [29]. Gold and titanium were described as conductors [30,31], silicon as a low-loss dielectric substrate, and polystyrene by a complex relative permittivity consistent with broadband THz characterization of polymers [32,33].

The 1.2–1.4 THz sensing sweep was selected to bracket the water-loaded fundamental resonance near 1.3 THz for the analyzed sensing configurations. The air-filled response is evaluated separately only to illustrate the effect of water loading using the same final sensor configuration; the 1.2–1.4 THz interval is not intended as a survey of higher-order modes outside the sensing band.

The polymer inclusions are treated as homogeneous continuum dielectrics using bulk THz permittivity values. This is a first-order constitutive approximation for subwavelength particles; possible size-dependent effective properties and interfacial or hydration layers are not resolved explicitly and may modify the effective dielectric contrast, particularly for the smallest inclusions.

The present simulations do not vary water temperature. Because the complex permittivity of water is temperature-dependent, an experimental implementation would require temperature control or temperature-specific baseline calibration. Salinity, dissolved species, and suspended impurities were not included in the present clean-water reference model. These constituents can modify both the real and imaginary parts of the complex permittivity of the aqueous matrix and may consequently affect the resonance frequency, resonance depth, linewidth, and particle-induced frequency shift. Therefore, environmental implementation would require calibration against a matrix-matched reference. A systematic quantitative assessment of these matrix effects is beyond the scope of the present study and is left for future work.

**Figure 4:**
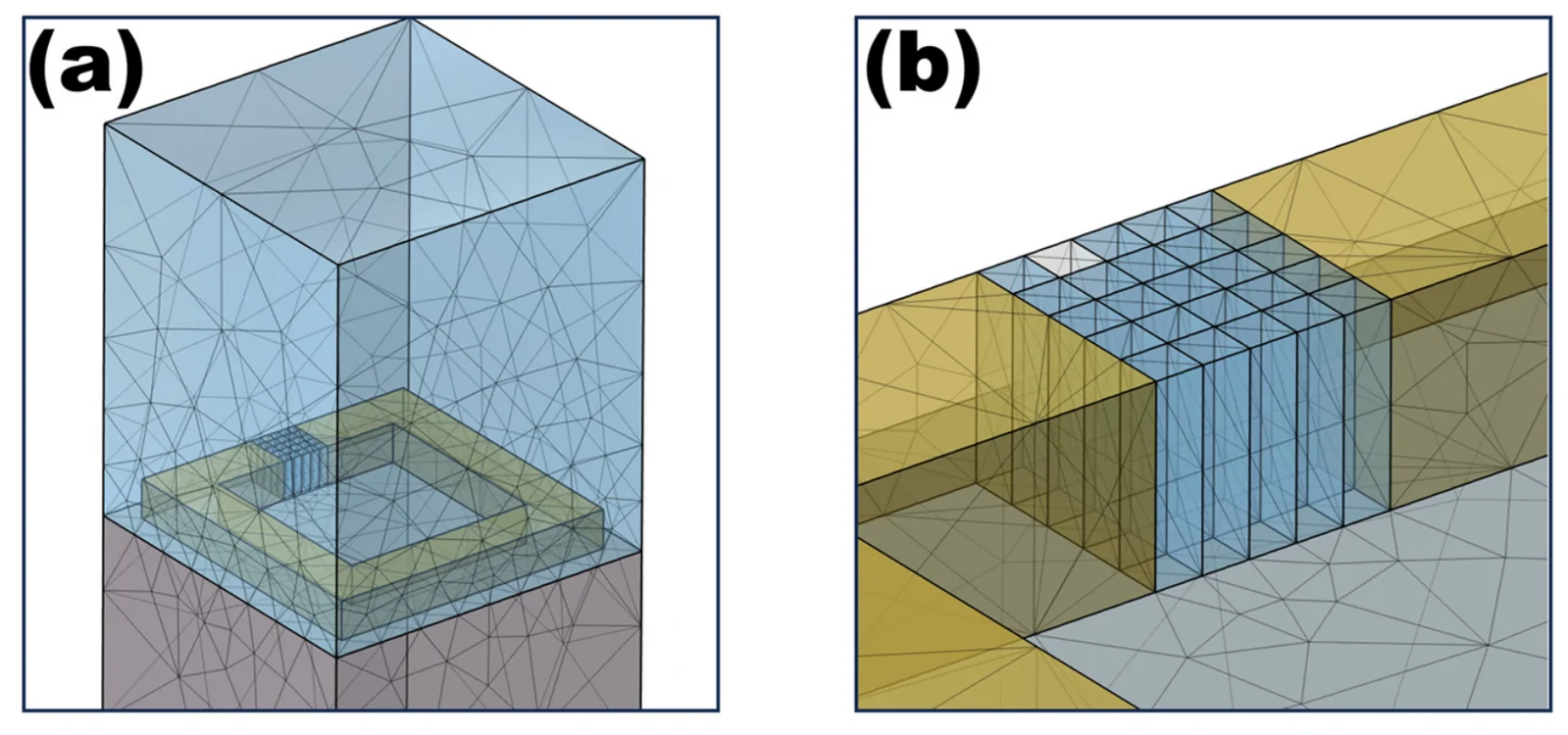
Finite-element discretization of the COMSOL unit-cell model: (**a**) complete tetrahedral mesh of the computational domain, including the silicon substrate, Ti/Au split-ring resonator, and water volume; (**b**) enlarged view of the active SRR-gap region, showing local mesh refinement within the sensing volume and along the material interfaces. The material colors follow Figure 3, while the overlaid dark lines indicate the finite-element mesh edges.

The tetrahedral volume mesh shown in Figure 4 is locally refined in the gap region and at material interfaces, where the field gradients are strongest. The final mesh configuration, used throughout the mapping, shape, and single-site simulations, comprises 7043 mesh vertices, 28,206 tetrahedral elements, 8619 triangular elements, and 1166 edge elements, with a minimum element quality of 0.2012 and an average element quality of 0.6736 (skewness measure). Each loaded variant is paired with a water-only reference computed using the same geometry, mesh configuration, and frequency range, so that the reported frequency shifts reflect the dielectric perturbation rather than discretization changes.

### 2.3. Analysis Methodology

For each simulation pair, the resonance frequency was identified from the transmission minimum. The parametric frequency sweep used a 1 GHz increment. To compare cases whose shifts are smaller than this increment, the resonance frequency was additionally estimated from a local quadratic fit through the three sampled points bracketing the *S*_21_ minimum:

(4) $$S_{21}(f)\;\approx \;A\;f^{2}\;+\;B\;f\;+\;C,\;f_{\mathrm{res},\mathrm{interp}}\;=\;\frac{-B}{2A}$$

The primary response metric is the resonance-frequency shift Δf = f_res,PS_ − f_res,water,_ where f_res,PS_ and f_res,water_ are the resonant frequencies of the loaded and matched water-reference configurations, respectively. The original 1 GHz sweep values were extracted by local quadratic interpolation and are reported to two decimal places; their precision was subsequently tested by the fine-sweep validation described below. Percentage differences are computed from unrounded fitted values.

To validate sub-GHz resonance extraction, a 14-cell subset of the 5 × 5 map was recalculated with a 0.01 GHz frequency increment using the same model and mesh settings. The subset spans the maximum- and minimum-response cells, the gap center, the edges and the corners. The error of the interpolation procedure was additionally quantified by resampling a 1 GHz grid from each dense sweep and comparing the interpolated position with the fine-sweep reference minimum. Fine-sweep calculations were also performed for spherical PS inclusions with r = 0.25, 0.50, and 0.75 µm. For the fine-sweep data, the resonance minimum was fitted locally within ±0.15 GHz, and robustness was checked using ±0.10 and ±0.20 GHz fitting windows.

### 2.4. Shape-Analysis Configuration

Motivated by the experimental observation that non-spherical polystyrene microbeads produce larger resonance shifts than spherical ones [20], the influence of inclusion shape was examined at strictly constant volume. A one-parameter family of equal-volume ellipsoidal inclusions was defined around a reference sphere of radius *r* = 1 µm; matched views of the two inclusion geometries are presented in Section 3.2. The ellipsoid semi-axes were (a, b, c) = (a, 1/a, 1) µm: a was aligned parallel to ax, b to ay, and c was aligned with the vertical axis corresponding to the resonator height. The vertical semi-axis was fixed at c = 1 µm, while a was swept from 0.80 to 1.25 µm in steps of 0.05 µm, and b was set to 1/a µm. Thus, every case had the volume of the reference sphere, and a = 1.00 µm reproduced the sphere. The cases a and 1/a describe the same solid rotated by 90° in the resonator plane, so the sweep probes both in-plane aspect ratio and orientation. The inclusion was centered in the active gap region, as in the radius study of Section 3.2.

Each shape case was solved over a frequency sweep of 1.2–1.4 THz with a 1 GHz increment. For each value of *a*, the polystyrene-filled configuration was paired with a water-only reference with exactly the same parametrized geometry, with the ellipsoidal domain retained but assigned the properties of water. Thus, the loaded and reference configurations within each pair were evaluated using the same geometry and finite-element mesh, ensuring that the extracted resonance shift for a given particle shape is not affected by mesh differences between the two material states. However, changing the aspect ratio modifies the ellipsoidal geometry and consequently requires remeshing between different values of *a*. Therefore, small differences between neighboring shape cases may include a discretization contribution and should not be overinterpreted.

Two mechanisms are expected to contribute to an orientation-dependent response at constant volume. First, in the quasi-static description of an ellipsoidal inclusion, the induced polarization depends on the depolarization factor along the excitation field, which differs between elongation parallel and perpendicular to the field. Second, the gap field of the SRR is strongly non-uniform, so the same elongated inclusion overlaps differently with the field hotspot depending on its in-plane orientation.

## 3. Results and Discussion

### 3.1. Transmission Response in Air and Water

**Figure 5:**
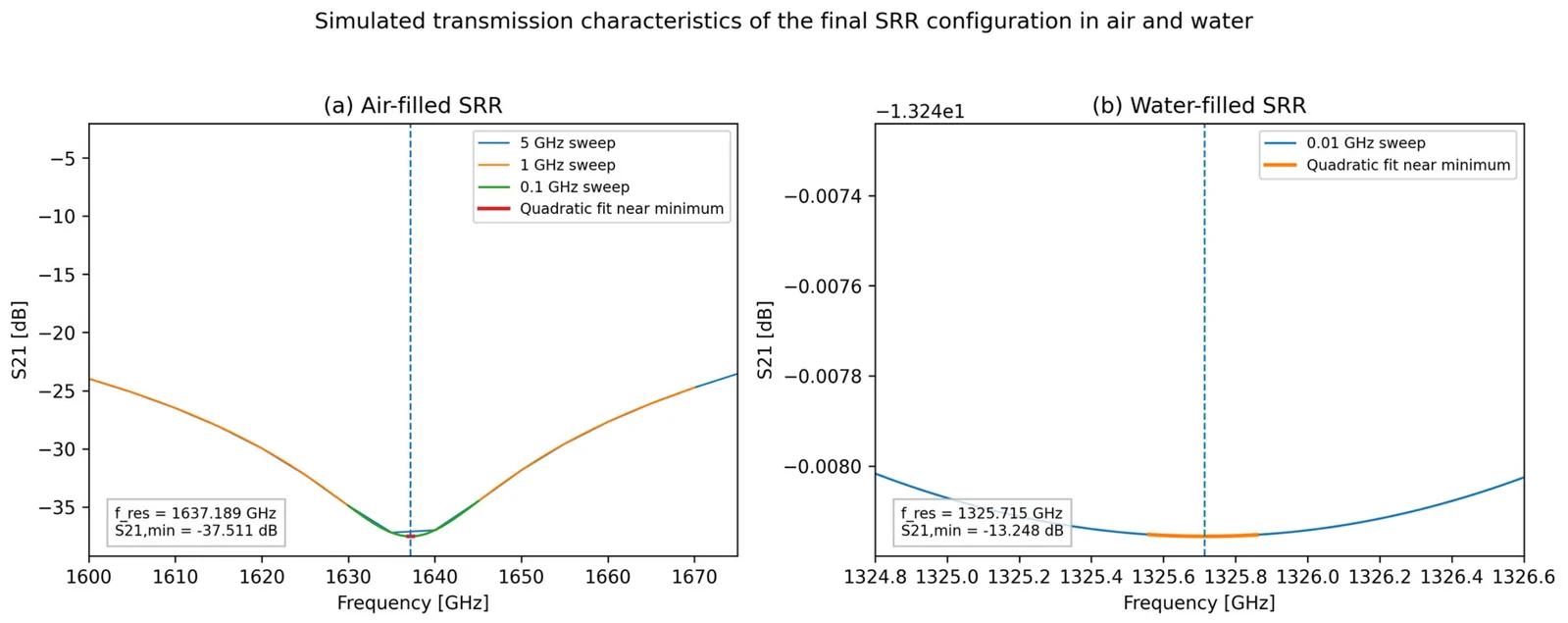
Simulated transmission characteristics *S*_21_(f) of the final SRR configuration in air and water. The extracted resonance minima are located at 1.63719 THz for the air-filled case and 1.32571 THz for the water-filled case. The dashed vertical lines indicate the fitted resonance minima in the air- and water-filled cases.

Figure 5 compares the transmission response of the final SRR configuration (*a*_x_ = 15 µm, *h*_Au_ = 2.00 µm) in air and in water, with all geometric, meshing, port, and boundary-condition settings held identical between the two simulations and only the surrounding medium changed. The air-filled resonance occurs at *f*_res_ = 1.63719 THz with *S*_21,min_ = −37.511 dB, whereas the verified water-only reference resonance is *f*_res_ = 1.32571 THz with *S*_21,min_ = −13.248 dB. This corresponds to a water-induced red shift of approximately 0.31147 THz. This water-loaded state is the common reference used throughout the subsequent particle-size, spatial-sensitivity, shape, and material perturbation analyses.

The fully water-filled configuration was chosen deliberately as a loss-heavy reference case in which the sensing response is evaluated without reducing field-water overlap by microfluidic confinement. It is therefore not presented as an optimized practical microfluidic architecture. In experimental implementations, reducing the water volume interacting with the resonant field and selectively concentrating particles in the active gap can improve measurement conditions.

Experimental studies by Cha et al. [21] and Park and Ahn [22] used spatial surface functionalization and, in the latter case, an aqueous microfluidic geometry to preferentially capture polystyrene particles in sensitive regions. The present numerical study complements those demonstrations by isolating position- and shape-dependent dielectric effects under fully immersed conditions; it does not model particle transport, capture kinetics, or selective surface chemistry.

### 3.2. Single-Site Response Versus Particle Radius

**Figure 6:**
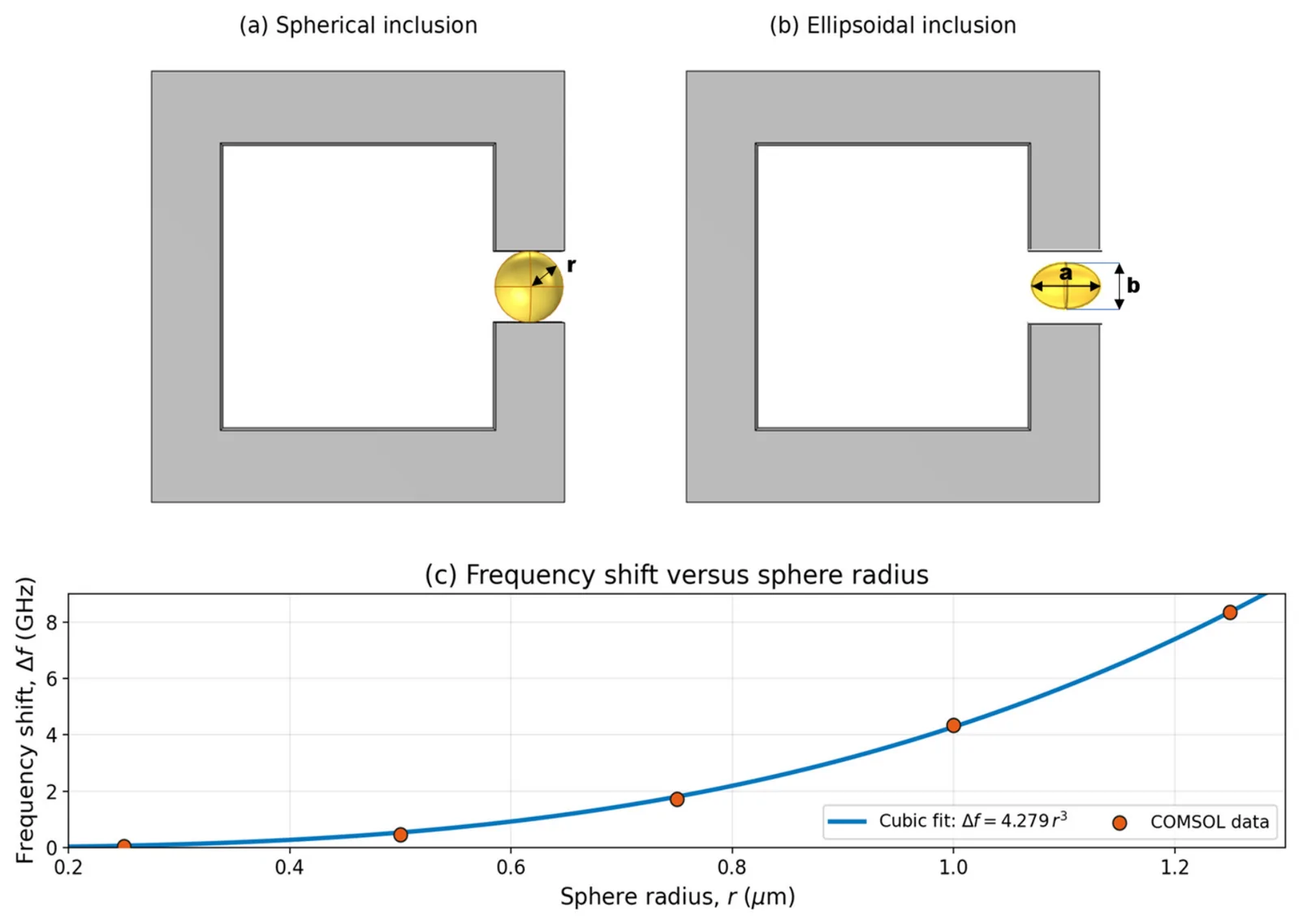
Inclusion geometries and single-particle radius response: matched full-cell top views of (**a**) a spherical inclusion and (**b**) an equal-volume ellipsoidal inclusion in the SRR gap; (**c**) frequency shift Δ*f* versus sphere radius *r* for the final configuration, with the constrained cubic fit Δ*f* = 4.2789*r*^3^ GHz (*r* in µm; *R*^2^ = 0.99966) and the data points. Panels (**a**,**b**) use the same resonator view, scale, and color palette. In panel (**c**), the markers denote the simulated frequency shifts and the solid curve denotes the constrained cubic fit.

Figure 6a,b compare matched top-view representations of the spherical and ellipsoidal inclusions in the same resonator cell and at identical display scale. Figure 6c shows the frequency shift produced by a single spherical polystyrene inclusion in the active gap as a function of its radius for the final configuration (*h*_Au_ = 2.00 µm). The interpolation-derived shifts were 0.05, 0.47, 1.72, 4.34, and 8.35 GHz for radii of 0.25, 0.50, 0.75, 1.00, and 1.25 µm, respectively. The constrained cubic fit Δ*f*(r) = *k* *r*^3^ yielded *k* = 4.2789 GHz/µm^3^ (*R*^2^ = 0.99966), consistent with the first-order volume scaling expected for a localized dielectric perturbation.

A 0.01 GHz local fine-sweep validation yielded Δf = 0.04968, 0.47206, and 1.71861 GHz for r = 0.25, 0.50, and 0.75 µm, respectively, confirming the corresponding values of 0.05, 0.47, and 1.72 GHz obtained from the original 1 GHz sweep with quadratic interpolation.

The water-loaded resonance has an estimated linewidth of approximately Γ = 71.15 GHz, corresponding to Q_loaded = 18.6. The smallest validated particle-induced shift of 49.68 MHz for r = 0.25 µm therefore represents approximately 0.07% of the resonance linewidth and should be regarded as experimentally demanding. The linewidth itself, however, does not directly define the detection limit. Fitting or interpolating the measured resonance line shape can improve the precision of the estimated resonance position relative to the frequency sampling interval, but it does not improve the intrinsic accuracy or stability of the measurement system. A frequency-domain TOPTICA TeraScan 1550 (TOPTICA Photonics SE, Gräfelfing, Germany) with the appropriate tuning-range extension provides a minimum frequency step below 10 MHz; however, practical detectability additionally depends on SNR, water attenuation, frequency repeatability, drift, baseline stability, and fitting uncertainty. Under a 3σ detection criterion, resolving the simulated 49.68 MHz shift would require a standard deviation of the experimentally determined resonance frequency below approximately 16.6 MHz. Since such repeatability has not yet been established experimentally under water-loaded conditions, an experimental LOD cannot be inferred from the deterministic simulations alone.

### 3.3. Spatial Sensitivity of the SRR Gap: 5 × 5 Map

For the spatial mapping, the active gap region was divided into a 5 × 5 grid of indexed loading regions, and each cell was individually filled with material representing polystyrene; every loaded case was compared with the common water-only reference of the final configuration (f_res,water_ = 1.32571 THz, S_21,min_ = −13.248 dB). Cell indices are given as (row, column), with (1, 1) at the top-left corner of the map.

The 5 × 5 discretization was selected to provide a spatial resolution of 0.5 µm across the 2.5 µm active-gap width, comparable to the diameter of the smallest particle considered in the radius study. This resolution was sufficient to capture the pronounced spatial variation in sensitivity across the SRR gap while maintaining a practical number of full three-dimensional simulations.

Each map cell spans approximately 0.5 × 0.5 × 2 µm^3^ and is used as a fixed volumetric dielectric probe. The map therefore represents the local dielectric sensitivity of the SRR gap to an equal-volume perturbation, not the trajectory or discrete position map of a physical particle. The probe volume is 0.50 µm^3^, close to the 0.524 µm^3^ volume of the r = 0.50 µm sphere used in the particle-radius study, which provides a useful scale comparison while preserving the distinction between the rectangular map probe and a real particle. Because each probe extends through the 2 µm vertical dimension, the reported 5 × 5 map is an in-plane indexing of the resonance response to a three-dimensional volumetric perturbation rather than a z-resolved surface-sensitivity map.

The spherical and ellipsoidal cases are likewise idealized fixed-position inclusions. Random transport, positional probability, and particle-wall interactions in a flowing sample are outside the present numerical model and are left to future microfluidic studies.

All 25 local PS perturbations produced positive Δ*f* values, as expected when lower-permittivity PS replaces part of the higher-permittivity water environment. The map spans 0.370–0.971 GHz, with a mean of 0.638 GHz and a standard deviation of 0.197 GHz. The maximum occurred at cell (1, 1), whereas the minimum occurred at cell (1, 3). Thus, nominally identical local perturbations produced a 2.62-fold difference in Δ*f* solely as a result of position within the active gap.

Fine-sweep validation on the 14-cell subset agreed closely with the original interpolation-derived values, with a mean absolute difference of 0.00030 GHz and a maximum absolute difference of 0.00068 GHz. The validation set was extended to 28 recomputed cases comprising the map subset, the five single-site material loadings, the three spheres, the three four-corner configurations and the three shape cases; every recomputed value agrees with the originally reported result to within 0.0051 GHz. The error of the interpolation procedure itself was quantified independently of any model or mesh difference: a 1 GHz grid was resampled from each dense sweep, the original three-point quadratic interpolation was applied to the resampled data, and the result was compared with the fine-sweep reference minimum of the same dense dataset. Over 71 such tests, spanning the map subset and the sphere series and with the resampling phase varied, the error was 0.00024 GHz RMS with a maximum of 0.00052 GHz. The extreme validated responses changed from 0.971 and 0.370 GHz to 0.97037 and 0.36989 GHz, respectively, leaving the reported sensitivity contrast unchanged at 2.62. Varying the local quadratic fitting window from ±0.10 to ±0.20 GHz changed the fitted resonance position by less than 0.00001 GHz.

**Figure 7:**
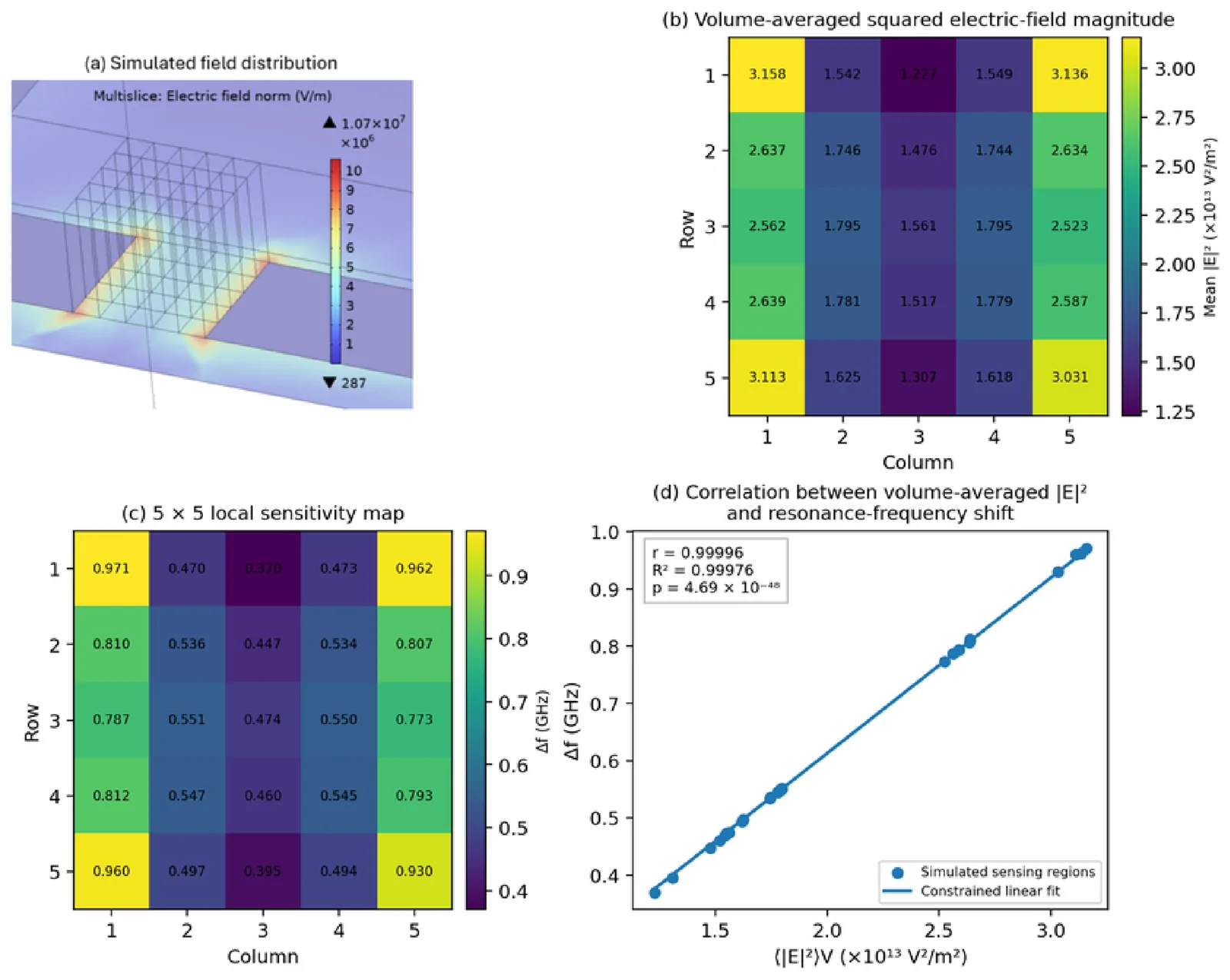
Quantitative relationship between the electric-field localization and the spatial sensitivity of the THz resonator for the reference configuration (a_x_ = 15 µm, hAu = 2.00 µm) at f_0_ = 1.3257 THz: (**a**) simulated electric-field distribution in the resonator gap for the unperturbed resonator, (**b**) 5 × 5 map of the volume-averaged squared electric-field magnitude, ⟨|E|^2^⟩_v_, evaluated over the same sensing subvolumes used in the particle-position analysis, (**c**) corresponding 5 × 5 local resonance-frequency-shift map, and (**d**) correlation between ⟨|E|^2^⟩_v_ and the resonance-frequency shift Δf. A linear regression physically constrained to pass through the origin yields R^2^ = 0.99976, while the Pearson correlation analysis gives r = 0.99996 and *p* = 4.69 × 10^−48^. The strong linear relationship confirms that the spatial variation in sensitivity is directly related to the local electric-field concentration. In panel (**d**), the markers denote the 25 simulated sensing regions and the solid line denotes the constrained linear regression.

The map corners consistently produced larger resonance-frequency shifts than the central column, as shown by the complete spatial distribution in Figure 7c. This trend is consistent with the representative simulated electric-field distribution in Figure 7a, which shows stronger field localization near the metallic gap edges and corners. To quantitatively verify this relationship, the electric-field distribution of the unperturbed reference resonator (a_x_ = 15 µm, hAu = 2.00 µm), without a dielectric inclusion, was evaluated at its reference resonance frequency of f_0_ = 1.3257 THz. The active gap region was divided into the same 25 subvolumes used for the 5 × 5 particle-position sensitivity analysis. For each sensing region, the volume-averaged squared electric-field magnitude was calculated as:

(5) $${\left\langle {\left|E\right|}^{2}\right\rangle }_{V_{\mathrm{ij}}}=(\frac{1}{V_{\mathrm{ij}}})\int_{V_{\mathrm{ij}}}{\left|E_{0}\right|}^{2}dV$$

Because all 25 sensing subvolumes have identical dimensions, the volume-averaged quantity is directly proportional to the corresponding integrated squared electric-field magnitude, ∫V_ij_|E_0_|^2^ dV. Therefore, both quantities provide the same relative spatial distribution of the electric-field concentration across the sensing area.

The resulting spatial distribution, shown in Figure 7b, ranges from 1.227 × 10^13^ to 3.158 × 10^13^ V^2^/m^2^ and closely follows the corresponding resonance-frequency-shift map. In particular, the highest values of ⟨|E|^2^⟩_v_ occur in the corner regions, where the largest frequency shifts are observed, whereas the central region exhibits both a lower electric-field concentration and a smaller resonance shift.

The quantitative relationship between these quantities is shown in Figure 7d. Analysis of all 25 sensing regions yields a Pearson correlation coefficient of r = 0.99996 and *p* = 4.69 × 10^−48^, demonstrating an almost perfectly linear relationship between the volume-averaged squared electric-field magnitude and the local resonance-frequency shift. Since the resonance-frequency shift should approach zero when the interaction of the dielectric perturbation with the local electric field approaches zero, the linear regression was physically constrained to pass through the origin. The resulting relationship is

(6) $$\Delta f=0.30653\left(\frac{{\langle {\left|E\right|}^{2}\rangle }_{V}}{10^{13}\frac{V^{2}}{m^{2}}}\right)GHz$$

with a coefficient of determination of R^2^ = 0.99976. The regression line in Figure 7d is displayed only over the range of electric-field values obtained from the 25 sensing regions, avoiding extrapolation outside the simulated data range.

These results confirm that the spatial variation in sensitivity is primarily associated with the non-uniform electric-field localization within the SRR gap. A local dielectric perturbation introduced into a region of stronger electric-field concentration produces a larger change in the effective resonator capacitance and, consequently, a larger resonance-frequency shift. The result also provides a direct design guideline for future microfluidic integration: particle trapping or preconcentration should preferentially target the high-field corner regions of the SRR gap rather than its geometric center.

### 3.4. Effect of Particle Shape at Constant Volume

**Figure 8:**
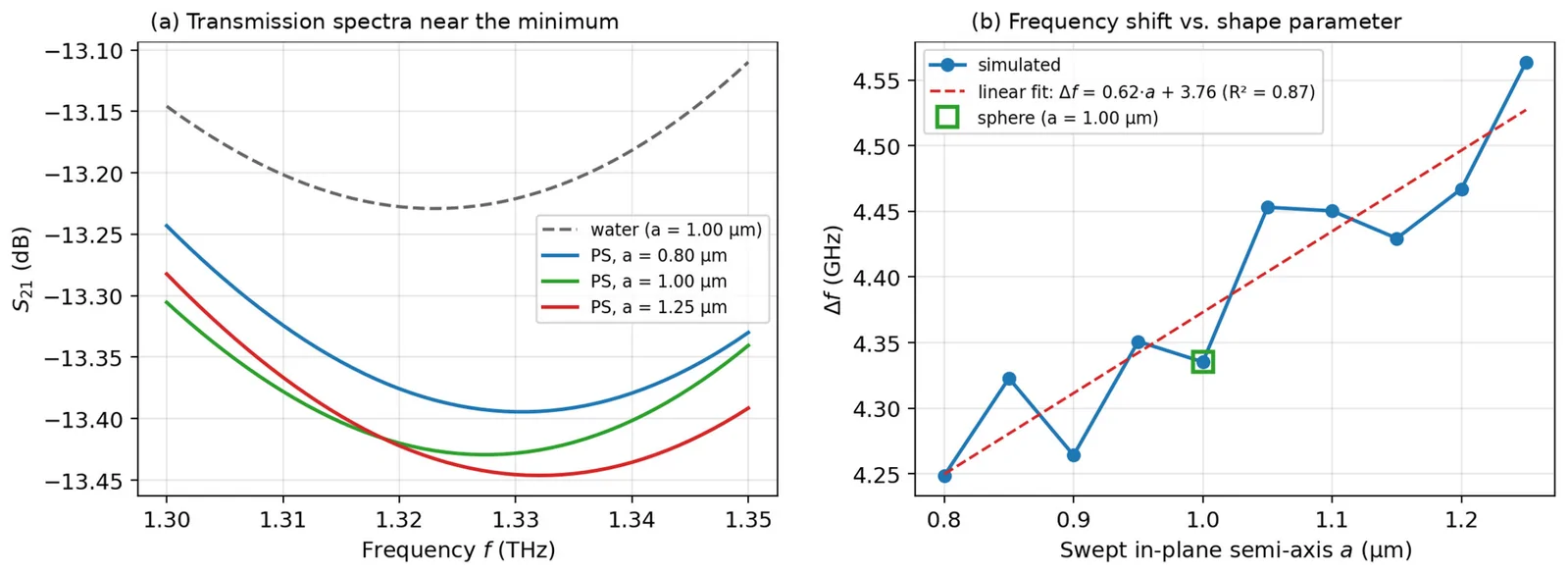
Equal-volume shape study: (**a**) *S*_21_ spectra near the transmission minimum for the extreme and spherical cases, together with the water-only reference; (**b**) Δ*f* as a function of the swept semi-axis *a*, with the fitted linear trend Δ*f* = 0.62*a* + 3.76 GHz (*R*^2^ = 0.87) and the spherical reference case marked. The corresponding ellipsoidal inclusion geometry is shown in Figure 6b. The cases *a* = 0.80 µm and *a* = 1.25 µm are 90° in-plane rotations of the same equal-volume solid.

Table 2 and Figure 8 summarize the frequency shifts produced by the equal-volume shape family defined in Section 2.4. All ten cases produced positive shifts between 4.25 and 4.56 GHz. The spherical case (*a* = 1.00 µm) yielded Δ*f* = 4.34 GHz, consistent to within 0.01 GHz with the corresponding spherical-inclusion result of the radius study in Section 3.2, which confirms the internal consistency of the two independently parametrized models.

The shift increases systematically with the semi-axis a: inclusions elongated along the b-direction (a = 0.80 µm, b = 1.25 µm) produced Δ*f* = 4.25 GHz, about 2.0% below the sphere, whereas the same solid elongated along the a-direction (a = 1.25 µm, b = 0.80 µm) produced Δ*f* = 4.56 GHz, about 5.3% above it. Over the tested range the trend is approximately linear in a, with a fitted slope of 0.62 GHz/µm (R^2^ = 0.87) and a total spread of 7.4% between the extreme cases. Individual point-to-point differences of up to about 0.06 GHz between neighboring cases should not be overinterpreted, because each aspect-ratio case involves a separately parametrized geometry and corresponding remeshing. The interpolation error quantified in Section 2.3 is far smaller; therefore, the robust result is the systematic trend across the complete ten-case series rather than any individual neighboring pair.

Because the extreme cases a = 0.80 µm and a = 1.25 µm describe the same solid rotated by 90° in the resonator plane, their difference provides a measure of the orientation sensitivity of the sensor: an identical elongated particle of identical volume, located at the same position, produces a different resonance shift depending on its in-plane orientation relative to the SRR gap. The vertical semi-axis remains fixed throughout the shape series, so this variation is associated exclusively with the in-plane geometry and orientation. Fine-sweep recalculation of the extreme and spherical cases yielded Δf = 4.2486, 4.3349 and 4.5637 GHz for a = 0.80, 1.00 and 1.25 µm, respectively, corresponding to an orientation contrast of 7.42% between the two extreme cases. The physical origin of this orientation dependence cannot, however, be assigned uniquely to a single mechanism. Changing the in-plane aspect ratio at constant particle volume simultaneously modifies the overlap of the inclusion with the strongly non-uniform electric field in the SRR gap and the shape-dependent depolarization response of the ellipsoidal inclusion. The latter is tensorial and depends on the orientation of the local electric field relative to the three principal axes of the ellipsoid. Because the present analysis uses the scalar squared electric-field magnitude |E|^2^, it does not provide the directional field information required to separate these contributions quantitatively. A rigorous decomposition would require component-resolved complex fields, volume-integrated field-overlap quantities for each particle geometry, and dedicated mesh-convergence analysis for the different parametrized ellipsoids. Accordingly, the observed 7.42% orientation dependence is interpreted here as the combined effect of non-uniform field overlap and shape-dependent depolarization, without assigning dominance to either mechanism.

This result remains consistent with previous experimental observations of shape-dependent THz sensing of polystyrene microbeads [20], while extending the analysis to a fully immersed water environment under a strict constant-volume constraint. Consequently, elongated microplastic particles with different in-plane orientations may produce a distribution of sensor responses even at identical volume and position, which should be considered in particle-preconcentration strategies and in the interpretation of measured resonance-shift distributions.

The position and shape studies were intentionally formulated as separate perturbations to isolate first-order effects: the map varies position while holding the rectangular probe geometry fixed, whereas the shape study varies aspect ratio and orientation at a fixed gap-center position and constant volume. The present data therefore do not demonstrate that position- and shape-induced variations are independent; their coupled response at off-center locations remains a separate multiparameter problem for future work.

### 3.5. Single-Site Response to Different Dielectric Loadings

**Figure 9:**
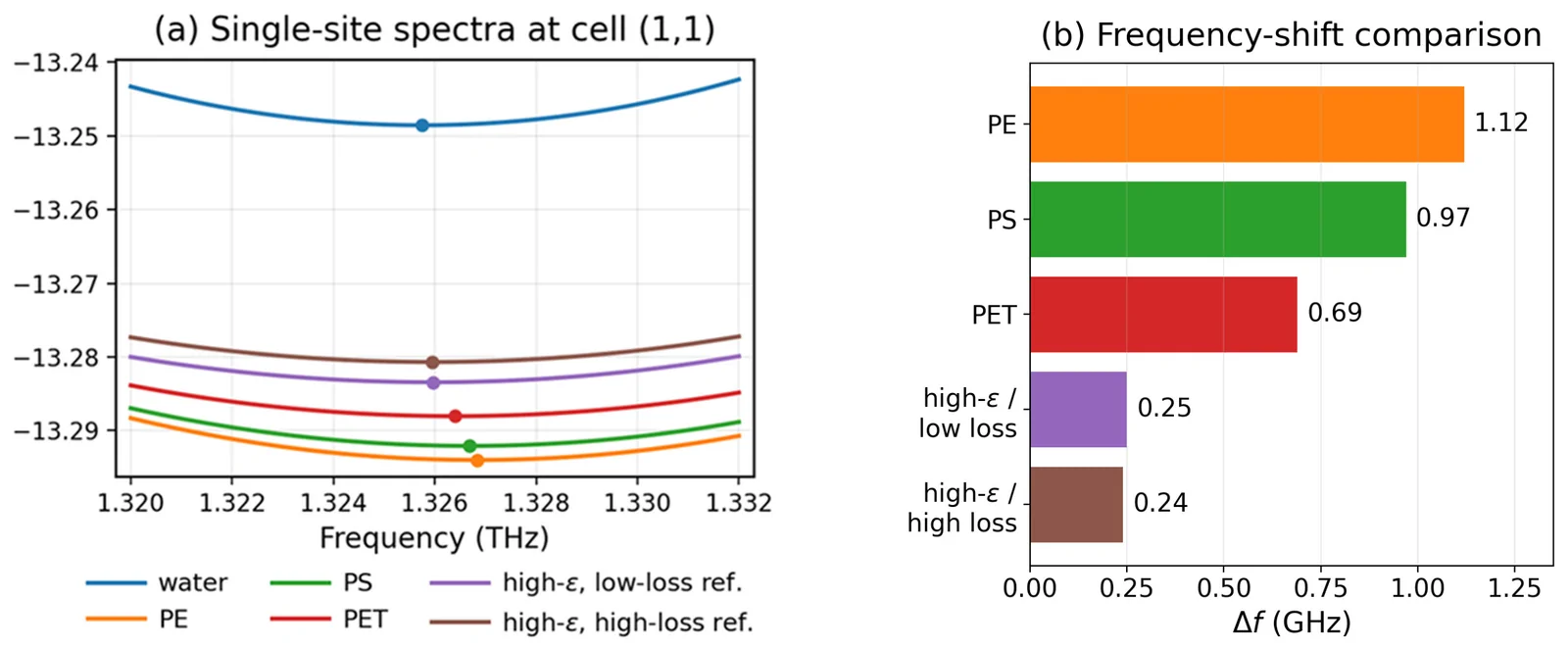
Single-site local-loading comparison at cell (1, 1): (**a**) *S*_21_ spectra around the transmission minimum for the five tested dielectric perturbations; (**b**) interpolation-derived Δ*f* values relative to the water-only reference. The two high-permittivity cases are synthetic controls used to distinguish permittivity and loss effects.

To determine whether the single-site response also discriminates analytes by their dielectric properties rather than merely by their presence, the same local-loading procedure used at the highest-sensitivity site (Section 3.3 and Section 3.4) was repeated for five materials spanning a range of permittivity and loss: PE, PS, PET, and two synthetic high-permittivity references introduced specifically to isolate the effect of dielectric loss at fixed permittivity. The resulting single-site responses are summarized in Table 3 and Figure 9. At the high-sensitivity cell (1, 1), Δ*f* decreased as the real part of the local dielectric permittivity increased from PE through PS and PET to the high-permittivity references. PE produced the largest shift, 1.12 GHz, followed by PS (0.97 GHz) and PET (0.69 GHz). The high-permittivity low-loss (High-ε LL) and high-permittivity high-loss (High-ε HL) references shared *ε*_r_ = 3.80 and produced nearly identical shifts, 0.25 and 0.24 GHz, respectively; their S_21_ minima differed by only about 0.003 dB. For this small local perturbation, the chosen tenfold loss change therefore did not yield a robustly separable amplitude response.

### 3.6. Four-Corner Perturbation and Approximate Additivity

**Figure 10:**
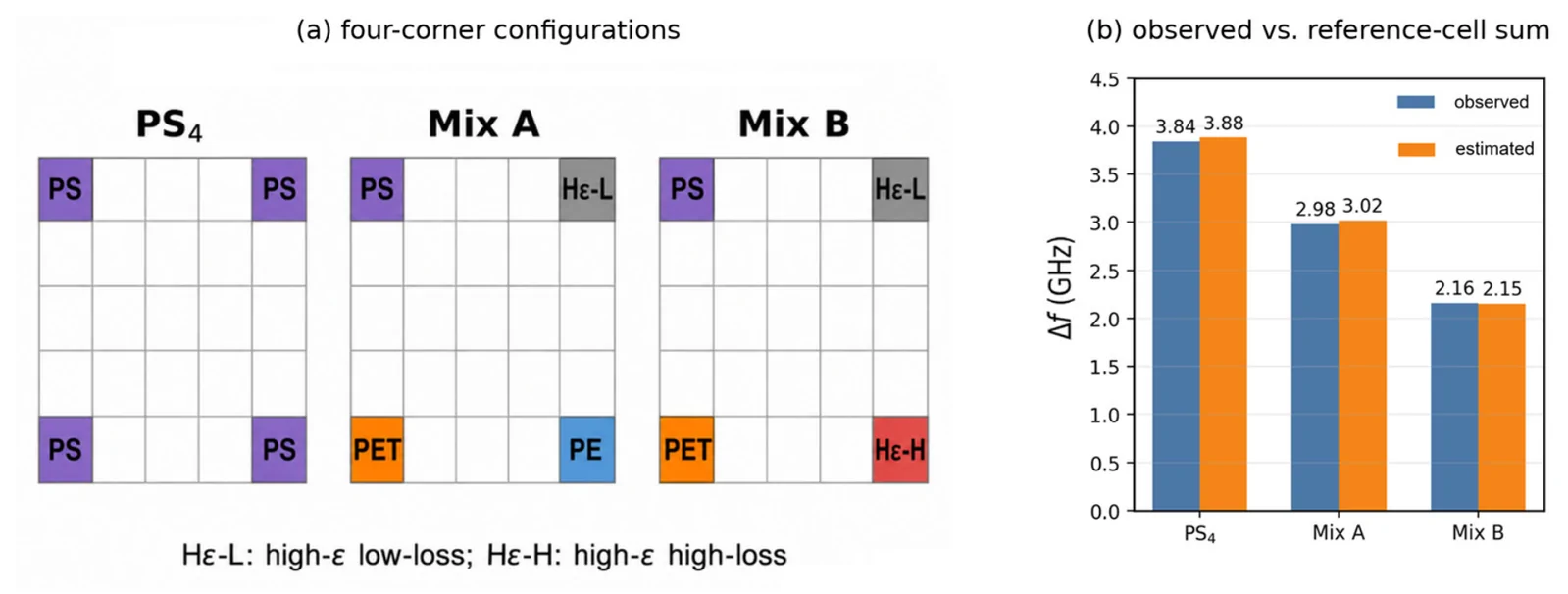
Four-corner local-loading study: (**a**) spatial arrangement of the three tested configurations on the 5 × 5 grid. PS_4_ contains polystyrene (PS) inclusions at all four corner cells. Mix A contains PS, the high-permittivity low-loss reference (Hε-L), polyethylene terephthalate (PET), and polyethylene (PE) at the upper-left, upper-right, lower-left, and lower-right corners, respectively. Mix B retains PS, Hε-L, and PET at the first three positions and replaces PE with the high-permittivity high-loss reference (Hε-H) at the lower-right corner; (**b**) observed Δ*f* values compared with additive estimates obtained by summing the corresponding single-site responses at cell (1, 1).

The preceding sections characterized the sensor response to a single local perturbation. A practical sample, however, may contain several particles distributed across the active gap, raising the question of how their individual contributions combine. To test this, three four-corner configurations were examined, in which the four highest-sensitivity corner cells of the 5 × 5 grid were loaded simultaneously (Figure 10a): PS_4_, with all four corners filled with polystyrene; Mix A, combining PS, PE, PET, and the high-ε low-loss reference; and Mix B, combining PS, PET, and the high-ε low-loss and high-ε high-loss references. In each case, the observed shift was compared with a reference-cell estimate formed by summing the corresponding single-site responses at cell (1, 1). The four-corner configurations showed approximately additive behavior relative to the reference-cell estimate. For PS_4_, the observed Δ*f* was 3.84 GHz, compared with a 3.88 GHz estimate obtained from 4× Δ*f*_PS_(1, 1), corresponding to −1.2%. For Mix A, the observed and estimated shifts were 2.98 and 3.02 GHz, respectively (−1.3%). For Mix B, the corresponding values were 2.16 and 2.15 GHz (+0.6%). In all three configurations, the observed shift agreed with the reference-cell sum to within 1.4%; percentage differences were computed from unrounded interpolation-derived values. The observed and reference-cell values are summarized in Table 4.

This observation is relevant to future preconcentration strategies, because particles placed at multiple high-sensitivity sites may yield a stronger response than a single particle. It should not be generalized as a universal additivity law or as a direct relation between frequency shift and particle concentration: the result remains conditional on the specific four-corner arrangements, the fixed geometry, and the absence of real sample-matrix effects.

## 4. Conclusions

This article presented a numerical characterization of the local dielectric sensitivity of a terahertz SRR metasurface operating in water. The 5 × 5 sensitivity map is the central result: identical rectangular polystyrene probe volumes produced shifts between 0.370 and 0.971 GHz depending on their position in the active gap, a 2.62-fold spread whose corner-dominated topology is consistent with the simulated field distribution. A representative 14-cell validation with a 0.01 GHz frequency step confirmed the original sub-GHz interpolation-derived map values. The equal-volume shape study showed an orientation dependence of about 7% at fixed volume and position. Single-site PE, PS, and PET responses followed the dielectric contrast against water, while the three tested four-corner arrangements showed approximately additive behavior relative to the reference-cell estimates within 1.4%; this observation is specific to those arrangements and is not a universal additivity law.

The main limitations define the scope of the conclusions. The spherical and ellipsoidal inclusions are idealized fixed-position objects, and no experimental limit of detection or chemical specificity is claimed. The 5 × 5 map represents the resonance response to fixed three-dimensional rectangular dielectric probes indexed by their in-plane position, rather than a particle trajectory or a z-resolved sensitivity map. Water is modeled as a clean dispersive medium without temperature variation, salinity, or matrix constituents, and the fully immersed configuration is a deliberately loss-heavy reference rather than an optimized microfluidic architecture. Within these bounds, the results provide numerical design guidance for future microfluidic preconcentration, matrix-specific calibration, coupled position-shape studies, and experimental validation.

Although the structure investigated in this study is passive and does not incorporate a tuning mechanism, the quantified spatial sensitivity distribution and the identified influence of local dielectric perturbations may provide a useful reference for future reconfigurable THz metasurface sensors. In such systems, resonance tunability could be introduced as an additional degree of freedom while preserving the localized sensing characteristics identified here. The present results should therefore be regarded as a static baseline for future studies of actively reconfigurable THz sensing architectures rather than as a demonstration of tunable operation.

## Figures and Tables

**Table 1:** Material parameters used in the numerical model.

Material	Model Description	Role
Water	dispersive *ε*(f) after Ellison [29]	reference medium
Gold	*σ* = 4.09 × 10^7^ S/m [30]	SRR resonator
Polystyrene	complex *ε*_r_ = 2.53(1 − *i*·0.0069) [32,33]	reference analyte
Titanium	*σ* ≈ 2.4 × 10^6^ S/m [31]; thickness 5 nm	adhesion layer
Silicon	*ε*_r_ ≈ 11.7, low-loss; thickness 520 µm	Substrate

**Table 2:** Frequency shifts for the equal-volume shape family, (*a*, *b*, *c*) = (*a*, 1/*a*, 1) µm. Every case has the volume of the *r* = 1 µm reference sphere and constant c. Percentage differences were computed from unrounded interpolation-derived values.

*a* (µm)	*b* = 1/*a* (µm)	In-Plane Aspect *a*/*b*	Δ*f* (GHz)	Difference vs. Sphere	
0.80		1.250	0.64	4.25	−2.0%
0.85		1.176	0.72	4.32	−0.3%
0.90		1.111	0.81	4.26	−1.6%
0.95		1.053	0.90	4.35	+0.4%
1.00 (sphere)		1.000	1.00	4.34	—
1.05		0.952	1.10	4.45	+2.7%
1.10		0.909	1.21	4.45	+2.7%
1.15		0.870	1.32	4.43	+2.2%
1.20		0.833	1.44	4.47	+3.0%
1.25		0.800	1.56	4.56	+5.3%

**Table 3:** Individual local dielectric-loading cases at cell (1, 1).

Case	*ε*_r_	tan *δ*	*f*_res,interp_ (THz)	Δ*f* (GHz)	*S*_21,min_ (dB)
PE	2.30	0.0040	1.326829	1.12	−13.29
PS	2.53	0.0069	1.326685	0.97	−13.29
PET	3.00	0.0200	1.326402	0.69	−13.29
High-*ε* LL ref.	3.80	0.0050	1.325962	0.25	−13.28
High-*ε* HL ref.	3.80	0.0500	1.325955	0.24	−13.28

**Table 4:** Four-corner local-loading configurations compared with the reference-cell sum of individual responses. Percentage differences were computed from unrounded values.

Configuration	Observed Δ*f* (GHz)	Reference-Cell Sum (GHz)	Difference
PS_4_	3.84	3.88	−1.2%
Mix A	2.98	3.02	−1.3%
Mix B	2.16	2.15	+0.6%

## Data Availability

The raw data supporting the conclusions of this article will be made available by the authors on request.
